# Family caregivers’ involvement in caring for frail older family members using welfare technology: a qualitative study of home care in transition

**DOI:** 10.1186/s12877-022-02890-2

**Published:** 2022-03-18

**Authors:** Heidi Snoen Glomsås, Ingrid Ruud Knutsen, Mariann Fossum, Karin Christiansen, Kristin Halvorsen

**Affiliations:** 1 Faculty of Health Sciences, Institute of Nursing and health promotion, Oslo Metropolitan University, Postbox 4, St. Olavs plass, N-0130 Oslo, Norway; 2 Faculty of Health and Sport Sciences, Institute of Health and Nursing Science, University of Agder, Postboks 422, N-4604 Kristiansand, Norway; 3 Faculty of Health Sciences, Research Centre for Health and Welfare Technology, VIA University College, Hedeager 2, DK-8200 Aarhus, Denmark

**Keywords:** Caregiver, Elderly, Ethics, Home care, Involvement, Qualitative research, Technology

## Abstract

**Background:**

Demographic, economic and organisational changes challenge home care services. Increased use of welfare technology and involvement of family members as co-producers of care are political initiatives to meet these challenges. However, these initiatives also involve ethical aspects.

**Method:**

The aim of this qualitative study was to explore family caregivers’ experience of involvement and possible ethical aspects of caring for frail older family members receiving home care services supported by welfare technology.

This study used a qualitative explorative and descriptive design within a phenomenological-hermeneutical approach. Sixteen interviews with eighteen family caregivers were conducted. The participants were sons, daughters, siblings and spouses of frail older people receiving home care services with the support of welfare technology. Data were analysed using reflexive thematic analysis. The COREQ checklist was used.

**Results:**

The analysis led to five main themes. First, the family caregivers’ experienced caring as meaningful but increasingly demanding concerning the changes in home care services. Second, they experienced a change in relationships, roles, tasks, and responsibilities related to more family involvement and the use of welfare technology. This also challenged their sense of autonomy. However, welfare technology helped them deal with responsibilities, especially safety. The family caregivers requested early involvement, dialogue for care decisions, more cooperation and support from health professionals. Third, the participants experienced that health professionals decided the conditions for co-production without discussion. Their need for information and knowledge about welfare technology were not met. Fourth, the family caregivers felt that the health professionals did not adequately recognise their unique knowledge of the care receiver and did not use this knowledge for customising the welfare technology to the care receiver and their families. Fifth, the family caregivers expressed concern about service and welfare technology inequality in home care services.

**Conclusions:**

Co-production in the involvement of family caregivers in care is still not an integral part of home care service. Welfare technology was appreciated, but the family caregivers called for early involvement to ensure successful and safe implementation and use. More attention needs to be given to ethical concerns about the change in relations, transfer of tasks and responsibility, and risk of inequality.

**Supplementary Information:**

The online version contains supplementary material available at 10.1186/s12877-022-02890-2.

## Introduction

During the past two decades, healthcare services have changed due to increasingly ageing populations, a shortage of health professionals and a transfer of specialised healthcare services to primary care [1–3]. The rationing potential of older people living longer in their homes receiving home care services is emphasised in health policy documents [4, 5]. To support services for older people, the importance of using welfare technology is underlined. Furthermore, health policy calls for family caregivers to co-produce care [2, 4, 6].

With the transformation of health care, home care is changing the service for older care receivers through increased use of welfare technology. More knowledge is needed about family caregivers’ experiences as co-producers of care using these technologies and their collaboration with the services. Investigation of ethical aspects of this complex interaction and co-production is underexplored and requires attention if the quality of care is to be ensured.

### Background

Older people living at home are often frail and need support from their family [2]. A central challenge in home care services seems to be that welfare technology is often introduced without involving the care receivers and their families in the process [7, 8]. Increased expectations of co-production of care and the use of welfare technology raise ethical concerns about how to balance responsibility and proper care, maintain trust and mutual respect for individuals’ autonomy, and secure equal access to welfare services.

### The role of family members

In Norway and the rest of Europe, there has been increasing awareness in health policy of family members’ vital contribution to the care of frail older people living at home [2, 4, 9]. Data from Europe show that 5.4% of women and 2.2% of men aged 18-64 have reduced their working hours or taken breaks from work of more than a month to take care of ill and older family members with disabilities [10].

Many family members wish to be involved in decision making and care planning in a collaborative practice with health professionals [11]. They can bring invaluable knowledge about the care receivers’ values, resources and needs [12, 13]. Their knowledge of the care receivers can improve home care services if used wisely [14, 15]. The ethics of care theory underlines family caregivers’ ability to recognise and respond to care receivers’ well-being and needs [16, 17]. However, numerous studies stress that the feeling of duty and responsibility for practical and psychological support can be challenging [14, 15, 18]. Family caregivers often perceive their role as lonely, exhausting and burdensome [18]. Challenges related to the power asymmetry between health professionals, family caregivers and care receivers, in terms of communicative strategies, have been identified [18, 19].

### User involvement and co-production

The trend towards more user involvement is expected to result in a demand for increased co-production. Co-production is receiving broad attention worldwide in healthcare. It can be seen as a step towards increased democratisation and the right to improve healthcare services [6, 20, 21].

The Co-production Network for Wales describes the term co-production as: ‘an asset-based approach to public services that enables people providing and people receiving services to share power and responsibility and work together in equal, reciprocal and caring relationships’ [22].

The public sector has embraced this involvement approach because of its potential to improve service quality and user satisfaction and reduce costs [6, 23]. Co-production occurs when people individually or collectively engage actively in delivering and designing the services they or their family members receive. Valuing knowledge from all parties involved while acknowledging each persons’ strengths are core principles in co-production and are inspired by common ethical values and principles [24, 25]. The emphasis is on peoples’ lives, not on the systems [22]. A positive attitude towards co-production and trust between family caregivers and health professionals are considered requirements for co-production of care [16, 26, 27]. However, there are ongoing debates about its definition and impact [28].

### Public home care services and implementation of welfare technology

There are differences in the availability and practical organisation of home care services for older people in Europe [29, 30]. In Norway, municipalities provide primary health and social care, including home care services. The home care service is organised by geographical areas and is an integral part of the healthcare service [31]. Taxes primarily finance the services, which are free of charge. All inhabitants of Norway have a legal right to receive healthcare regardless of age, gender and socio-economic status [32]. Equal access to health services is an essential ethical principle [33]. The Norwegian healthcare model is based on solidarity, focuses on universal civil rights, and is part of the Scandinavian or Nordic welfare model. However, the ageing population, immigration, globalisation and limited resources challenge the model [9]. Despite the basic premise of equal access to services, we see in Norway and Europe that access to and use of healthcare services vary among population groups and are related to income and education level [34–36].

We have chosen the Scandinavian umbrella term ‘welfare technology’ to describe and name technological solutions used to support older people living at home [37]. Welfare technology can also function as a support for family caregivers [38]. One of the most used definition of welfare technology in Norway is:

‘*Welfare technology is primarily technological assistance that improves the safety, security, social participation, mobility and physical and cultural activity, and strengthens the ability of individuals to fend for themselves in everyday life despite illness and social, mental or physical disability. Welfare technology can also act as support to their families and otherwise help to improve availability, resource utilisation and quality of service provision. Welfare technological solutions in many cases can prevent the need for services or institutionalisation*’ ([39], p 99). Translation by Hole [40].

Kamp et al. [37] point out that the term is broad and loosely defined, covering a wide array of technologies. In international literature on healthcare technology, terms like assistive technology, telecare, telehealth and e-health are used, but the dividing lines between them seem to be blurred [41, 42]. To increase quality, save time and cut costs, welfare technology is expected to be an integral part of primary healthcare in Norway [43]. However, welfare technology affects the lives of care receivers and family caregivers and therefore involves empirical, practical, and ethical issues related to the introduction of welfare technology.

Many frail older people want to live at home for as long as possible, supported by home care services and family caregivers. Welfare technology can help to make this possible [8, 44]. Welfare technology solutions are being implemented to improve safety and care quality for care receivers and family caregivers [2, 37]. They may reduce the stress and strain experienced by some family caregivers [38]. However, family caregivers’ views and experiences of welfare technology and their involvement in implementation and daily use have been poorly documented [45].

### Ethical aspects of family caregivers’ involvement and welfare technology

Involvement and the increased use of welfare technology include ethical aspects for care receivers and their families [46, 47]. Some ethical implications of welfare technologies have been examined and discussed, such as implications for privacy, freedom and autonomy of care receivers [46, 48, 49]. However, little consideration has been given to the implications for the involvement of family caregivers when the use of welfare technology is increased.

To understand family caregivers’ experience and the values at stake, we examine the importance of personal relationships and responsibility inspired by the ethics of care theory [16, 17]. The values of responsibility, concern and attachment forming care are based on personal relationships. Personal attitudes, such as respect for the other and the desire to provide care, are central [16]. Still, family members can also perceive care as an obligation and an added burden in their daily lives [16]. Hence, the feeling of obligation and added responsibility can challenge the family caregivers sense of autonomy and feeling of agency. According to Beauchamp and Childress [33], we ought to have the freedom to plan and live our lives according to our desires, beliefs and preferences. However, as Tronto [17] points out, people are not fully autonomous since we are interdependent, social beings relying on others for advice and support. There can be a tension between respecting a persons’ autonomy and the principle of benevolence and care for the vulnerable other. This dilemma might not only be apparent in the care for the frail family member but might also characterise the relationship between the family carers and the health professionals as well.

Another essential aspect of relationships between people is trust. Trust is based on the understanding that another person or persons will have honourable intentions with their actions. For there to be trust between people, such understanding must be mutual. Care is both a value and a practice and must be based on mutual concern, respect and trust [17].

## Methods

### Aim

The aim of this qualitative study was to explore family caregivers’ experience of involvement and possible ethical aspects of caring for frail older family members receiving home care services supported by welfare technology.

### Research design and philosophical approach

This study used a qualitative explorative and descriptive design. A qualitative design entails gathering data related to the participants’ perceptions and reflections [50]. The study was based on individual interviews of family caregivers, which were recorded and transcribed, thus producing texts that we could interpret [51, 52]. We were particularly interested in family caregivers’ experiences of changes in relationships, roles, responsibilities and tasks and ethical aspects of their involvement in caring for their older family member using welfare technology.

A phenomenological-hermeneutical approach was chosen to capture and understand the richness, complexity and individuality of the participants’ experiences [50, 51]. We focused on the family caregivers’ experiences in real-life circumstances regarding actions, attitudes and relationships. We were inspired by van Manen’s phenomenology when exploring and attempting to understand the essential meaning of the phenomena when the family caregivers’ expressed their lived experiences and ethically difficult situations in home care [53]. However, the research method is also hermeneutical since it is based on text interpretation [54]. Our pre-understanding of the parts of the text emerged and led to new understandings in a circular process.

### Research context

Family caregivers in six municipalities in south-eastern Norway participated in the study. It included both urban and rural areas. The municipality with the smallest population has approximately 1800 inhabitants and covers 500 km^2^, while the largest has about 86,000 inhabitants and covers 410 km^2^ [55].

The municipalities were obliged by the national authorities to implement welfare technology in their daily home care services but were at different stages. Digital door locks, digital medicine dispensers, patient alarms (both analogue and digital) with and without an integrated global positioning system (GPS), watches with GPS, stove guard, window and door sensors and digital calendars and planners supported the frail older family members received care in this study.

### Recruitment

The management of the home care service had knowledge of the family caregivers who were actively involved in caring for their family members where different types of welfare technology were used. The management contacted, informed, and recruited potential participants by combining their knowledge of the family caregivers with the inclusion criteria and our request for both genders to be represented, different ages and relation to the caregiver. The management did not state how many family members were asked and if any refused to participate; they only informed about the number of participants that accepted the invitation. The management collected signed informed consent forms and passed them to the first author before the interviews. When the management invited potential participants, they told the family members that the study focused on their experiences of user involvement and the care receivers use of welfare technology. A definition of welfare technology was included in the consent form: ‘With welfare technology, we think of technical solutions that are adapted to users’ needs, for example, safety alarms, door and windows sensors, GPS trackers, digital door locks and various types of robots such as medication dispensers.’ The family caregivers also received an information letter about the study to give to the care receiver and asked for their oral consent before the interviews. The caregivers were asked to withdraw if the care receiver did not want them to participate in the study. The first author contacted the participants by phone to schedule the interviews.

To be included in the study as a family caregiver, a person had to be mentioned as the closest family member in the care receivers’ electronic medical record. In addition, the caregivers’ family members who received home care services had to have used welfare technology for at least six months and be over 65 years old. Eighteen family caregivers participated: eight men and ten women aged 54 to 77 years (average 64). The participants consisted of two spouses, six sons, nine daughters and one sibling.

### Interviews

The authors developed a qualitative semi-structured interview guide (Table 1). An essential prerequisite for the successful introduction and use of welfare technology is mutual respect and collaboration with care receivers and their families. To gain a deeper understanding of the family caregivers real-life experiences, some questions focused especially on involvement, relations, information, and knowledge exchange with the health professionals since this may indirectly impact the use of welfare technology.

**Table 1:** Questions in the interview guide for family caregivers

	Questions
1	Could you tell me how your mother/father/sister/brother/husband/wife got the welfare technology? - Could you describe your involvement in the process?
2	Could you describe how your mother/father /sister/brother/husband/wife uses the welfare technology and whether you give him/her assistance in any way?
3	Could you describe how you find health professionals’ interest in your experiences and wishes for your mother/father/sister/brother/husband/wife?
4	Could you describe your experience of information exchange with the home care services?
5	Could you describe your experience of cooperating with home care services?
6	Could you describe how you feel about giving care and whether it has affected your relationship with the care receiver?
7	Do you have any concerns about your family member using welfare technology? -If so, could you describe them?
8	What are your thoughts on how your involvement in the care could be improved?
9	Can you describe what you think could improve the quality of home care services in general?

Sixteen individual interviews were planned and arranged. However, two extra siblings asked to participate in two of the interviews. For that reason, eighteen family caregivers participated in the study. The first author conducted all the interviews. She met the participants for the first time and were the only person present besides the participants in the interviews. The first author is a registered nurse and doctoral student and has previous experience in individual interviews and qualitative methods. A semi-structured interview guide was used (Table 1). Notes were taken for the analysis both during and after the interviews, as Brinkmann and Kvale (2015) recommend. During all the interviews, attention was paid to the participants’ experiences and how these were expressed. Information that emerged during the interviews was regularly summed up to validate or clarify the participants’ meaning. After 16 interviews, the first author stopped collecting data since the authors agreed that satisfactory saturation had been achieved.

The family members chose the time and place of the interviews. Nine interviews were conducted by telephone. Seven were conducted face-to-face, two in private homes, and the remaining five in quiet public places. The interviews lasted 20-62 min (average 35 min). They were recorded digitally and transcribed verbatim but de-identified. The first author transcribed five of the interviews and a professional transcriber eleven.

### Data analysis

Thematic analysis inspired by Braun, Clarke, Hayfield, and Terry [52] was employed to analyse themes and patterns in the dataset, as shown in Table 2.

**Table 2:** The six phases of thematic analysis [52]

Phase	Description of the process
1 Familiarisation	We read and re-read the dataset and took notes through a curious approach to what was interesting in the data and to notice possibilities, connections, and quirks, which may add depth and nuance to our later coding.
2 Generating codes	Essential characteristics of the data that might be relevant to answering the aim of the study were identified. We organised data around similar meanings. By generating codes, we got a sense of the participants’ experiences of involvement, welfare technology and ethical aspects.
3 Searching and construction themes	We examined the codes systematically and identified patterns of meaning, developing potential themes from the analytical work and ‘tested it out’ concerning the aim.
4 Reviewing themes	All the themes were discussed and revised to avoid overlaps and to understand how each of the themes was related to each other. They were checked across the whole data set to determine if they reflected the data and the aim.
5 Defining and naming themes	We explored how well the themes worked together and separately and finished by defining and naming the final themes.
6 Producing the report	The last step was the selection of examples, preparing and writing this article.

The analyses were manually carried out. All the authors were involved in all parts of the analysis. Table 3 svisualise how three different quotes were condensed to the same main theme.

**Table 3:** Example of the coding- and analyse process

Main theme	Preliminary themes	Examples of codes	Examples of quotes
Recognising complementary forms of knowledge.	Recognise the family caregivers’ knowledge and information needs of welfare technology. Recognise family caregivers’ knowledge of the caregivers. Access to health personnels’ knowledge of welfare technology.	Not know what to ask about Know the challenges in everyday life. Need information and knowledge of welfare technology.	*We do not know what we can ask about…and maybe the missing link is. Because we can see how complicated things are in everyday life ... we do not know if there is any technology that can help our mother* [7]
		Have in-depth knowledge about the care receiver. Health professionals do not have time to get to know the care receiver.	*No one knows our mother well, except us then. No one from the home care service has been with her for so long that they know her* [2].
		Health professionals know what kind of welfare technology is available. Welfare technology as support to administering of medication.	*The nurse in home care service asked if we would try a medicine dispenser because my wife uses many pills, and I have to make sure she gets it* [10].

The results were presented and discussed in an external advisory group for the PhD project of which this study is a part. One caregiver and one care receiver in this group were recruited from two pensioners associations. One caregiver was recruited from a group of patients and next of kin via the National Association for Public Health. Reflections that emerged during meetings in the advisory group did not produce any immediate changes to the analysis but rather confirmed the analytical reflections.

The Consolidated Criteria for Reporting Qualitative research (COREQ) checklist for reporting qualitative studies was used (Additional file 1).

### Ethical considerations

The principles for medical research stated in the Declaration of Helsinki [56] were followed. All participants received verbal and written information about the study and signed an informed consent form before the interviews. Information was provided about the possibility to withdraw from the study before the data were analysed. Confidentiality and anonymity were assured and safeguarded. Guidelines for storing research material were followed. Participation was voluntary, and no financial compensation was given. The study was registered with the Norwegian Centre for Research Data (NSD), reference number 473910. The Norwegian Regional Committee for Medical and Health Research Ethics, South-Eastern Norway (REK south-east), considered the study. They waived the ethical approval, reference number 2018/2462, in view of the procedures during the data collection and the nature of the study.

## Results

Eighteen adult family caregivers with close and long-term relations with their frail older family members participated. The participants had varied backgrounds regarding their health, social and economic status. The family caregivers had different needs, knowledge and experiences of involvement and welfare technology. The two spouses lived with their wives and were retired from work. Most of the daughters, sons and the sister lived with their own families, and several worked full-time.

Five main themes were identified in the analysis. First, the family caregivers’ experience of caring as meaningful but demanding. Support and discussion with the health professions were expressed as important for caregiving. Second is the experience of changing roles, tasks, and responsibilities to follow up the care receivers and how the welfare technology worked. Third, the family caregivers’ experience of health professionals decided the conditions for collaboration without dialogue. The health professionals did not explore whether the family caregiver had sufficient information and knowledge to follow up on the care receivers’ use of technology. Fourth, the need to recognise complementary forms of knowledge. The family caregivers pointed out that they ought to be involved early to adapt the technology to care receivers. Fifth and finally, the family caregivers’ concern about inequality related to their knowledge and the care receivers’ finances concerning the access to services and welfare technology.

In this section, we have added the interview number of the participants in parentheses at the end of the quotation.

### Caring is perceived as meaningful but also demanding

The family caregivers’ close relationship and emotional attachment to the care receiver contributed to their wish to give care and perceive it as meaningful. Several family members spent most of their spare time assisting the care receiver. Family carers responded very positively to situations where health professionals discussed their perception of the conditions for care and offered increased services and welfare technology in times of need. Although this happened rarely, it provided the family caregivers with a sense of safety and renewed energy for continuing the care.

‘I am very concerned about whether I can manage to handle my wife at home. …… we were invited to a meeting where they said they could offer her a place at the day centre three days a week. Getting that offer before I even started to ask for it was a nice gesture’ [1].

Nevertheless, most participants found caregiving to be demanding and exhausting. These feelings were particularly strong among those who did not lived with the care receiver and worked full-time. When the family caregivers could not spend sufficient time with their older family members to care for them properly, various emotions such as pain and guilt transpired. The participants expressed frustration and tension building up when health professionals did not understand the constraints they were working under. This often resulted in feelings of anger, sadness and helplessness. Several family caregivers said that most health professionals did not seem to care about those feelings and showed no particular interest or empathy with their situation.

‘When we are not there enough, it feels like we are not caring for our mother properly. It hurts to feel like that. My mother thinks she does not need any help from home care services because she has six children. She relies on us. Now it is our turn’ [9].

### Changed roles, tasks and responsibilities

The family caregivers felt a high degree of responsibility for the well-being and safety of their parents, siblings or spouses. They also said that using welfare technology freed up time and supported them in creating a safe environment and dealing with the anxiety of not being available 24 h a day.

‘It was a relief when mother got that medication dispenser and that she has the safety alarm. It’s crucial to relax a little and know that mother ... that someone will come and help her if we’re not close by. It’s crucial that we feel safe then’ [10].

Most of the family caregivers wanted to be involved in the care and the care receivers’ use of welfare technology. The results indicated only a few established routines and wide variations in how the home care services informed caregivers and followed up general needs and special needs connected to welfare technology. There were no regular collaboration meetings, although these were requested by many participants. Family caregivers usually took the initiative to contact home care services to discuss the care receivers’ situation, the need for care and how welfare technology could support them and the care receivers.

‘I would like to get involved. I always try to make it possible for my mother to have a good life in her flat for as long as possible. So, I want to get as much information as I can’ [10].

The family caregivers respected the family members’ desire for autonomy and to live relatively independently in their own homes for as long as possible. However, the participants had to assess and respond to signs of frailty such as cognitive decline. They stressed the importance of having a close dialogue with health professionals about changes in the health status of care receivers requiring adjustments to the use of welfare technology. There were examples of care receivers who no longer remembered that they had a particular technology or had forgotten how to handle it properly. Hence, new safety concerns arose and had to be addressed regularly.

‘She has a safety alarm but does not know how to use it. She does not understand… she no longer thinks about the fact that she might need help’ [9].

The participants expressed concern about the rationalisation of home care services, such as fewer visits due to increased allocation and implementation of welfare technology for the older care receivers. One example was a care receiver who only received help in administering medication. When the care receiver got a medication dispenser, the number of visits from health professionals was reduced from daily visits to only once every fortnight. In these situations, the family caregivers felt more responsibility was added to their normal duties regarding follow-up and reporting back on the patients’ needs, health changes and potential health issues.

### Health professionals decided the conditions for collaboration

In several situations, the participants found that health professionals decided the conditions for their collaboration and took their contribution and efforts for granted. They, therefore, felt that the power relationship was asymmetrical.

‘I had to take time off from work because I had to come at a time that suited the health professionals. It was completely wrong for me. I have already spent so much time there to assist the home care service’ [11].

Several family caregivers expressed frustration that changes in home care services primarily seemed to transfer tasks and responsibilities from health professionals to them. They felt they did not receive the necessary information and had no dialogue and discussion before tasks and responsibilities were transferred. As the quote below shows, one family caregiver was not even consulted by the health professional before she was responsible for explaining and repeating information to her mother about the use of welfare technology.

‘They just said to her that it would be a lot of information, but I would explain to her after they had left’ [2].

### Recognising complementary forms of knowledge

The long-term personal relationship between family carers and care receivers gave a unique insight into the care receivers’ specific values, needs and demands. This knowledge could be essential for the wise implementation of welfare technology in a particular context. Several participants felt that the health professionals showed little respect for this kind of knowledge and did not ask for it.

‘No one knows our mother well, except us then. No one from the home care service has been with her for so long that they know her’ [2].

The availability of a named contact person in the home care service, whom one could easily reach and communicate with, was considered highly important for co-production. One participant emphasised that it was much easier to find suitable technological solutions quickly and meet the care receivers’ needs if the health personnel knew the caregivers and care receiver.

Several participants found it frustrating not knowing what welfare technologies were available on the market, what they could apply for, and the procurement process.

‘We do not know what we can ask about…and that's maybe where the missing link is. Because we can see how problematic things are in everyday life ... we do not know if there is any technology that can help our mother’ [7].

The participants emphasised the importance of receiving information and becoming more actively involved early in the process concerning proper allocation and implementation of welfare technology to ensure that it met the care receivers’ needs. Home care services implementing welfare technology without dialogue with care receivers and family caregivers about material circumstances and daily practices and routines could decrease the likelihood of appropriate use and raise safety concerns.

‘The medication dispenser was initially put in my mother’s living room by the healthcare professionals. But she needs to reach the medication while she is still in bed’ [11].

An important factor for mutual understanding and cooperation is trust. When information from home care services about welfare technologies or services was considered unclear, inconsistent or unreliable, it created frustration and distrust among family caregivers. One example was two daughters who had received different information from separate health professionals about the services available. This made it impossible to navigate appropriately between the information and arguments provided to reach reasonable and well-informed decisions.

### Concerns about inequality

Several participants reflected on the close relationship between the level of services received, the number of follow-up visits from home care service, the availability of welfare technology and well-educated family members with insights or interests in welfare technology advocating for the care receiver.

‘I am an engineer by education, so I am all for implementing welfare technology. I read up on everything about it. I always like to ask, and I think that is the reason why we got the technology’ [1].

The participants mentioned that in some situations the home care services did not offer the requested technology. Instead, the health professionals recommended the care receiver or the family caregiver to buy or rent it themselves. However, this was not possible for all families; the cost of buying or renting technology devices was a matter of concern. Since home care services are financed through taxes in Norway, some participants felt that such additional costs placed an unfair burden on them or the care receivers.

## Discussion

The family members’ experience of involvement and welfare technology was influenced by various factors such as knowledge, background, living conditions, and the health status of those involved. Involvement as co-production implies practical and moral acts, where people must relate to each other and work together in equal, reciprocal and caring relationships*.* The consequences and ethical aspects of the changes in home care service and what is considered essential for family caregivers in this context will be discussed.

### Caring as meaningful but also demanding

The ethics of care theory assumes that we are relational, dependent and vulnerable beings, relying on each other for care and support. Familial, social and historical contexts are essential in care [16, 17]. This can partly explain why long-term relationships and emotional ties play a crucial role in family members’ wishes and sense of responsibility to care for parents, siblings and spouses. The close relationship provides an experience of caring as valuable to the family caregiver, as emphasised by Held [57]. Family caregivers are, in principle, autonomous and free to live their lives according to their desires, beliefs and preferences [33]. However, they are dependent on and shaped by their relationships with and expectations of care receivers, which affect the feeling of autonomy. Held [57] supports the notion that family caregivers can never be fully autonomous but understand themselves as acting in relation to care receivers and health policy requirements.

An integral part of health professionals’ work is the ethical focus on doing right for care receivers and family caregivers [33]. However, it may be questioned whether family caregivers perceive the attitudes and practices of health professionals as the best practice.

for the family caregiver. There is no doubt that the participants in the present study found that their care burden could be overwhelming when tasks and responsibilities were transferred from health professionals without considering their strengths, weaknesses and life situations. Ethical concerns are raised when responsibility and tasks are transferred to family members without considering their ability to take responsibility and risk potential adverse health consequences. Previous studies have shown an increased risk of depression, anxiety and sleep disorders due to excessive strain on family caregivers [58, 59]. Although the participants in this study did not report such health problems, several reported high levels of stress and exhaustion.

In general, the family caregivers appreciated welfare technology since it contributed to security and independence for the care receivers and themselves. For that reason, it reduced some of the care burdens, which other studies also support [38, 60]. Nevertheless, welfare technology was also experienced demanding since health professionals expected family caregivers to follow up information and the care receivers use. This shows some of the double-sidedness of using welfare technology. It both eases and add to the burdens of the family caregivers.

Even though providing good care is essential for many family caregivers, they pointed out the importance of balancing responsibility for the care receiver with taking care of themselves. Self-care is now more important with the expectation of increased involvement and responsibility. Plöthner [61] recommends focusing on early identification of caregivers’ needs and preferences and close follow-up from health professionals to reduce the care burden and enable caregivers to bear the responsibility over time.

### Changed roles, tasks and responsibilities

As supported by other studies, welfare technology can decrease family caregivers’ burden and make it easier to deal with the responsibility, especially in terms of safety and freedom [62, 63]. However, family caregivers’ close attention and ability to follow up on any problems is essential to identify how well the welfare technology works for the care receiver and make changes if needed. It is also essential to identify changes in cognitive functioning and assess whether the care receiver can no longer handle the technology, as pointed out in our study and other studies [64, 65]. The family caregivers found it demanding to make such assessments independently, with limited support from health professionals. This again highlights the urgency of developing a sustainable co-production approach.

When health professionals expect family caregivers to act on information, this means an extra task and responsibility, which may be felt like a forced order and limit family caregivers’ autonomy. There is also an increased risk of misunderstandings and misinformation if the information must go through several channels before reaching the care receiver. Studies of health professionals have shown that lack of competence could lead to incorrect use of welfare technology [66] and uncertainty and resistance [67]. This could clearly also apply to many family caregivers. Therefore, health professionals should be very careful about the types of information and responsibility to be transferred to family caregivers, especially if the results could have adverse consequences for the care receiver.

While some family caregivers would have been happy to take on more responsibilities and perform additional tasks and roles, this was not true of all of them. An important fact that all parties must take seriously is that some family caregivers are frail themselves and do not have the capacity to perform the expected tasks, especially not when the tasks and responsibilities increase. Younger family caregivers might also have particular needs and wishes that have to be addressed. Many have full-time work besides caring for young children. They may not be able to be involved as much as health professionals or the care receiver request. If there is no proper exchange of information and clarification of the nature and scope of caregivers’ involvement, this can create serious tensions between the parties.

If family caregivers’ needs and capacity to be involved are ignored, and tasks and responsibilities are just transferred without dialogue about individual family caregivers’ health and life situation, the caregivers might find the care burden excessive and withdraw from the caregiving role. In line with Plöthner et al. [61] and Tønnessen et al. [14], we recommend regularly discussing tasks and experiences to ensure that family members have the necessary skills and knowledge about welfare technology and time and energy to provide care.

### Health professionals decided the conditions for collaboration

Health professionals’ attitudes and willingness to share power and responsibility with family caregivers and give them a voice are among the most important factors for successful co-production [26]. When health professionals stipulate conditions for collaboration with family caregivers, this indicates an unequal and non-mutual relationship. The participants seemed to agree that involvement should take place through partnership, and develop and mature through mutual dialogue and negotiation of power between health professionals and family caregivers, as suggested by Gheduzzi et al. [68]. This way of working and thinking enhances care receivers’ satisfaction and quality of health [28].

Since older care receivers depend on help from others, they transfer power and trust to family caregivers or health professionals to provide satisfactory care. Trust is a mutual understanding of intentions and expectations [16]. It is an example of a value inherent in an ethics of care since good caring relationships depend on it. Trust is also essential for optimal use of welfare technology and co-production of care. Health professionals show their values, attitudes and desires to involve family caregivers through their actions. Our study shows that several family caregivers felt vulnerable and relied on support, information, acknowledgement and close follow-up from health professionals to cope with the challenges of caregiving. However, they felt health professionals did not show them respect and recognised their unique knowledge or efforts. In several situations, the participants felt their responsibility for the care receivers use of welfare technology was taken for granted by the health professionals.

The participants provided examples of situations where they did not feel adequately acknowledged and respected as caregivers. Unfortunately, the combination of low trust and lack of mutual respect between the parties might lead to low satisfaction with the healthcare service, thus threatening the continuity of care and co-production. In line with Gheduzzi et al. [68], we recommend that health professionals change their attitude and work towards co-production in care.

### Recognising complementary forms of knowledge

One of the main purposes of co-production is to recognise the value of multiple kinds of knowledge and use this to improve the organisation of health care services and provide optimal care to care receivers [26]. Family caregivers know the care receivers far better than health professionals, at least in terms of their preferences, values and goals. However, health professionals hold invaluable medical knowledge developed through training, education and clinical practice. Ris et al. [11] suggested that recognising the complementary forms of knowledge and expertise between family caregivers and health professionals is essential for family caregivers’ involvement. It is also vital to use family caregivers’ knowledge to select welfare technology and adapt it to the individual users’ needs and coping capacity. Respect for the family caregivers’ knowledge requires recognising different knowledge and avoiding paternalistic domination from the health professionals, as the ethics of care highlight [57]. Health professionals need to listen with interest to family caregivers, recognise them as partners in care, and show respect for their knowledge. Insight into each other’s specific competencies is required [69, 70]. Further, co-production also requires sufficient time to cooperate and insight into the philosophy and methodology of co-production [20]. The importance of working within a co-production framework was addressed indirectly by the participants when they reflected on the benefits and challenges of implementing welfare technology. The participants agreed that welfare technology must be tailored to the individual user to be used as intended [64, 71]. Much will be gained if family caregivers, who know the needs and interests of the care receiver, are invited into a dialogue with health professionals about identifying and allocating suitable technology. This is particularly important if the care receiver is technologically illiterate or suffering from cognitive decline. If the health professional is open and responsive to the unique insights and contributions of the family caregivers, the utilisation of welfare technology and quality of care is likely to improve.

### Inequality in care

Equality as a moral principle enshrined in human rights [72] and is an essential tenet of the modern welfare state. The Norwegian Patient Rights Act states that all patients have an equal right to health care [32]. The fact that some family caregivers found a close association between their knowledge and engagement with the home care received and the availability of welfare technology suggests vulnerability and inequality. It is legally and ethically problematic if access to welfare technology and various services depends on the care receivers’ financial situation and on family caregivers’ knowledge or ability to stand up for the care receiver. There is a risk that the most vulnerable people and those without a family will receive lower service quality.

A review by Scott Kruse et al. [73] identified cost as one of the main barriers to adopting welfare technology. If some people cannot afford to buy or rent the equipment, there is a risk of inequality. It also makes it less likely that the technology will be used by many families to maximum benefit. Fewer users of the technology may threaten the policy initiatives to meet challenges in home care with increased use of welfare technology [37].

## Limitations and methodological concerns

With 18 participants, the results provide a limited picture of family caregivers’ experiences of involvement, welfare technology and possible ethical aspects. Further, our participants may have had a higher socio-economic status than average since they agreed to participate in the study. For these reasons, the results cannot be generalised.

Moreover, we are aware that results of interviews can differ according to whether they are conducted in peoples’ homes or public places, and with or without other people nearby [51]. The two interviews where two siblings asked to be present affected the interview situation and challenged our plan of using only individual interviews. However, with their presence, the data in those two interviews were more nuanced.

Further, individual face-to-face interviews often become more personal and deeper than telephone interviews [51]. Additionally, telephone interviews do not allow us to observe body language. The duration of the interviews and some of the participants’ short answers may have resulted in less substantial content than desirable. Nevertheless, the qualitative data provided a rich picture of family caregivers’ experiences.

## Conclusions

The family caregivers felt that they had a moral responsibility to observe and respond to care receivers’ needs and use of welfare technology. The feeling of obligation to provide care to family members and health professionals’ expectation of increased involvement challenged the family caregivers’ autonomy. However, welfare technology supported the participants in creating a safe environment and freeing up time. Still, welfare technology also made new tasks and responsibilities for information and followed up of the care receiver.

Equal and fair access to healthcare service is a democratic ideal, which means equal access to services and welfare technology for all people. It seems essential that the transfer of tasks, roles and responsibilities is clarified and adapted to family caregivers’ capacity and opportunity for co-production in care for their older family members. Health professionals’ must give attention to family caregivers’ living situations and provide adequate support to reduce the care burden and enable them to bear the responsibility of care over time. Reliable information and trust are vital for family caregivers to co-produce care in a close relationship with home care services. Sharing power and responsibility and respecting mutual knowledge must be paramount when the goal is to improve the quality of home care service. However, the family caregivers experienced that home care services were not prepared for their involvement as active and equal partners in co-production when implementing and using welfare technology.

## Supplementary Information

**Additional file 1.** COREQ (Consolidated Criteria for Reporting Qualitative Studies) checklist.

## Data Availability

Data can be available from the corresponding author for an appropriate purpose within the study framework. The data is in Norwegian. Permission from NSD and the participants have only been granted to use data for this current study.

## Abbreviations

COREQ checklist: The Consolidated Criteria for Reporting Qualitative research checklist
PhD project: Doctoral project
NSD: The Norwegian Centre for Research Data
REK: The Norwegian Regional Committees for Medical and Health Research
GPS: Global positioning system

## References

[1] European Commission. The 2021 ageing report. Underlying assumptions and projection methodologies 2020. Available from: https://ec.europa.eu/info/sites/info/files/economy-finance/ip142_en.pdf.

[2] Eurostat. Ageing Europe. Looking at the lives of older people in the EU Luxembourg2020. Available from: https://ec.europa.eu/eurostat/documents/3217494/11478057/KS-02-20-655-EN-N.pdf/9b09606c-d4e8-4c33-63d2-3b20d5c19c91?t=1604055531000.

[3] European Commission. Green paper on ageing. Fostering solidarity and responsibility between generations Brussels, Belgium2021. Available from: https://ec.europa.eu/info/sites/info/files/1_en_act_part1_v8_0.pdf.

[4] Ministry of health and care services. National Health and Hospital Plan 2020–2023 Oslo2020. Available from: https://www.regjeringen.no/contentassets/95eec808f0434acf942fca449ca35386/engb/pdfs/stm201920200007000engpdfs.pdf.

[5] World Health Organization. The growing need for home health care for the elderly Egyp: WHO Library Cataloguing in Publication Data; 2015. Available from:https://apps.who.int/iris/bitstream/handle/10665/326801/EMROPUB_2015_EN_1901.pdf?sequence=1&isAllowed=y.

[6] European Commission. Co-production. Enhancing the role of citizens in governance and service delivery, Luxenburg2018. Available from: https://ec.europa.eu/european-social-fundplus/system/files/2021-06/TD4-Co-production%20-%20enhancing%20the%20role%20of%20citizens%20in%20governance%20and%20service%20delivery.pdf.

[7] Frennert S, Östlund B. Narrative review: Welfare Technologies in Eldercare. Nord j sci tech stud. 2018;6(1):21-34. 10.1353/sym.2003.0023.

[8] Bjørkquist C, Forss M, Samuelsen F. Collaborative challenges in the use of telecare. Scand J Caring Sci. 2019;33(1):93-101. 10.1111/scs.12605. 10.1111/scs.12605 30113071

[9] Norwegian Ministry of Health and Care Services. Care Plan 2020: The Norwegian Government’s plan for the care services field for 2015–2020 Oslo2015. Available from: https://www.regjeringen.no/contentassets/af2a24858c8340edaf78a77e2fbe9cb7/careplan2020_eng.pdf.

[10] Eurostat. Reconciliation between work and family life Labour Force Survey (LFS) ad-hoc module 2018 Luxembourg2019. Available from: https://ec.europa.eu/eurostat/documents/7870049/10159907/KS-FT-19-006-ENN.pdf/49232e21-106f-308f-8dc6-fd75b30ded86.

[11] Ris I, Schnepp W, Mahrer Imhof R. An integrative review on family caregivers' involvement in care of home-dwelling elderly. Health Soc Care Community. 2019;27(3):e95-e111. 10.1111/hsc.12663. 10.1111/hsc.12663 30307685

[12] Manias E, Bucknall T, Hughes C, Jorm C, Woodward-Kron R. Family involvement in managing medications of older patients across transitions of care: a systematic review. BMC Geriatr. 2019;19(1):95. 10.1186/s12877-019-1102-6. 10.1186/s12877-019-1102-6 PMC6441224 30925899

[13] Wilson SJ, Martire LM, Sliwinski MJ. Daily spousal responsiveness predicts longer-term trajectories of patients’ physical function. Psychol Sci. 2017;28(6):786-97. 10.1177/0956797617697444. 10.1177/0956797617697444 PMC5467745 28459650

[14] Tønnessen S, Solvoll B-A, Brinchmann BS. Ethical challenges related to next of kin - nursing staffs’ perspective. Nurs Ethics. 2016;23(7):804-14. 10.1177/0969733015584965. 10.1177/0969733015584965 26002940

[15] Callaghan S. A collaborative partnership between patient and caregiver. Nurs Stand. (Royal College of Nursing (Great Britain) : 1987). 2012;26:33. 10.7748/ns.26.49.33.s51 10.7748/ns.26.49.33.s51 28072253

[16] Held V (2006). Ethics of care.

[17] Tronto JC. Moral Boundaries: A Political Argument for an Ethic of Care. New York: Routledge; 1993.

[18] Søvde BE, Hovland G, Ullebust B, Råholm MB. Struggling for a dignifying care: experiences of being next of kin to patients in home health care. Scand J Caring Sci. 2019;33(2):409-16. 10.1111/scs.12638. 10.1111/scs.12638 30604881

[19] Lilleheie I, Debesay J, Bye A, Bergland A. Experiences of elderly patients regarding participation in their hospital discharge: a qualitative metasummary. BMJ Open. 2019;9(11):1-16. 10.1136/bmjopen-2018-025789. 10.1136/bmjopen-2018-025789 PMC6858187 31685492

[20] Vennik FD, van de Bovenkamp HM, Putters K, Grit KJ. Co-production in healthcare: rhetoric and practice. Int Rev Adm Sci. 2015;82(1):150-68. 10.1177/0020852315570553.

[21] Hamann J, Heres S. Why and How Family Caregivers Should Participate in Shared Decision Making in Mental Health. Psychiatr Serv. 2019;70(5):418-21. 10.1176/appi.ps.201800362. 10.1176/appi.ps.201800362 30784381

[22] The Coproduction and Involvement Network for Wales. What is co-production? 2021. Available from: https://copronet.wales/. Accessed 15 Nov 2021.

[23] Loeffler E, Bovaird T, Ongaro E, Van Thiel S (2018). From participation to co-production: widening and deepening the contributions of citizens to public services and outcomes.

[24] Staniszewska S, Hickey G, Coutts P, Thurman B, Coldham T. Co-production: a kind revolution. Res. Involv. 2022;8(1):4. 10.1186/s40900-022-00340-2. 10.1186/s40900-022-00340-2 PMC8817643 35123585

[25] International Council of Nurses. The ICN Code of Ethics for Nurses Geneva, Switzerland2021. Available from: https://www.icn.ch/system/files/2021-10/ICN_Code-of-Ethics_EN_Web_0.pdf.

[26] Social Care Institute for Excellence. Attitudes towards co-production. London; 2019. Available from: https://www.scie.org.uk/files/co-production/week/2019/co-pro-survey-2019.pdf.

[27] Birkhäuer J, Gaab J, Kossowsky J, Hasler S, Krummenacher P, Werner C, et al. Trust in the health care professional and health outcome: A meta-analysis. PLoS One. 2017;12(2):1-13. 10.1371/journal.pone.0170988. 10.1371/journal.pone.0170988 PMC5295692 28170443

[28] Voorberg WH, Bekkers VJJM, Tummers LG. A systematic review of co-creation and co-production: Embarking on the social innovation journey. Public Manag. Rev. 2015;17(9):1333-57. 10.1080/14719037.2014.930505.

[29] Genet N, Boerma W, Kroneman M, Hutchinson A, Saltman RB. Home care across Europe: Current structure and future challenges. Copenhagen: WHO Regional Office for Europe; 2012. Available from: https://www.euro.who.int/__data/assets/pdf_file/0008/181799/e96757.pdf.

[30] van der Roest HG, van Eenoo L, van Lier LI, Onder G, Garms-Homolová V, Smit JH, et al. Development of a novel benchmark method to identify and characterize best practices in home care across six European countries: design, baseline, and rationale of the IBenC project. BMC Health Serv Res. 2019;19(1):310. 10.1186/s12913-019-4109-y. 10.1186/s12913-019-4109-y PMC6521361 31092244

[31] Lov om kommunale helse-og omsorgstjenester [The Act Relating to Municipal Health and Care Services]. LOV-2011-06-24-30. Helse- og omsorgsdepartementet. 2011. Available from: https://lovdata.no/dokument/NL/lov/2011-06-24-30

[32] Lov om pasient- og brukerrettigheter [The Act Relating to Patients’ Rights]. LOV-1999-07-02-63. Helse- og omsorgsdepartementet. 1999. Available from: https://lovdata.no/dokument/NL/lov/1999-07-02-63//dokument/NL/lov/1999-07-02-63/.

[33] Beauchamp T, Childress J. Principles of Biomedical Ethics. 8th Edition. ed. Oxford og New York: Oxford University Press; 2013.

[34] Wiborg ØN, Hansen MN. The Scandinavian model during increasing inequality: Recent trends in educational attainment, earnings and wealth among Norwegian siblings. Res Soc Stratif Mobil. 2018;56:53-63. 10.1016/j.rssm.2018.06.006.

[35] World Health Organization. Regional office for Europe. Healthy, prosperous lives for all: the European Health Equity Status Report Copenhagen, Denmark 2019. Available from: https://www.euro.who.int/en/publications/abstracts/health-equity-status-report-2019.

[36] OECD. Understanding the socio-economic divide in Europe: The OECD Centre for Opportunity and Equality; 2017. Available from: http://projects.mcrit.com/foresightlibrary/attachments/article/1173/cope-divide-europe-2017-background-report.pdf.

[37] Kamp A, Obstfelder A, Andersson K. Welfare Technologies in Care Work. Nord. J. Work. Life Stud. 2019;9(S5):1-12. 10.18291/njwls.v9iS5.112692.

[38] Davies A, Brini S, Hirani S, Gathercole R, Forsyth K, Henderson C, et al. The impact of assistive technology on burden and psychological well-being in informal caregivers of people with dementia (ATTILA Study). Alzheimers Dement (NY). 2020;6(1):e12064. 10.1002/trc2.12064. 10.1002/trc2.12064 PMC7539670 33043107

[39] Norwegian Ministry of Health and Care Services. Innovasjon i omsorg [Innovation in the Care Services]. Helse- og omsorgsdepartementet; 2011. NOU 2011:11.

[40] Hole G. What is essential for successful implemention of welfare technology? Work process simplification is essential for success in welfare technology 2017. Available from: http://www.dr-glennhole.org/what-is-essential-forsuccessful-implementation-of-welfare-technology-work-process-simplification-is-essential-for-success-in-welfaretechnology/. Accessed 1 Nov 2021.

[41] Solli H, Bjørk IT, Hvalvik S, Hellesø R. Principle-based analysis of the concept of telecare. J Adv Nurs. 2012;12:2802-15. 10.1111/j.1365-2648.2012.06038.x 10.1111/j.1365-2648.2012.06038.x 22607115

[42] Barrett D, Thorpe J, Goodwin N (2014). Examining perspectives on Telecare: factors influencing adoption, implementation, and usage. Smart Homecare Technol TeleHealth.

[43] Norwegian Centre for E-health Research. Effects of welfare technology in Norway: Norwegian Centre for E-health Research. 2021. Available from:https://ehealthresearch.no/en/projects/effects-of-welfare-technology-in-norway. Accessed 15 Nov 2021.

[44] Glomsås HS, Knutsen IR, Fossum M, Halvorsen K. User involvement in the implementation of welfare technology in home care services: The experience of health professionals—A qualitative study. J Clin Nurs. 2020;29(21-22):4007-19. 10.1111/jocn.15424. 10.1111/jocn.15424 33463827

[45] Smithard D. Family carers/next-of-kin perceptions of home-care technology: a review. Smart homecare technol. telehealth. 2014;2014:45. 10.2147/SHTT.S42675.

[46] Hofmann B. Ethical challenges with welfare technology: a review of the literature. Sci Eng Ethics. 2013;19(2):389-406. 10.1007/s11948-011-9348-1 10.1007/s11948-011-9348-1 22218998

[47] Mort M, Roberts C, Pols J, Domenech M, Moser I. Ethical implications of home telecare for older people: a framework derived from a multisited participative study. Health Expect. 2015;18(3):438-49. 10.1111/hex.12109. 10.1111/hex.12109 PMC5060789 23914810

[48] Bennett B, McDonald F, Beattie E, Carney T, Freckelton I, White B, et al. Assistive technologies for people with dementia: ethical considerations. Bull World Health Organ. 2017;95(11):749-55. 10.2471/blt.16.187484. 10.2471/BLT.16.187484 PMC5677608 29147055

[49] Sánchez V, Taylor I, Bing-Jonsson P. Ethics of smart house welfare technology for older adults: a systematic litteature review. Int J Technol AssessHealth Care. 2017;33:691-9. 10.1017/S0266462317000964. 10.1017/S0266462317000964 29151393

[50] Bowling A (2014). Research methods in health: investigating health and health 4ed.

[51] Brinkmann S, Kvale S (2015). InterViews, Learning the craft of qualitative research interviewing, Third Edition ed.

[52] Braun V, Clarke V, Hayfield N, Terry G, Liamputtong P (2019). Thematic analysis. Handbook of research methods in health social sciences.

[53] van Manen M (1990). Researching lived experience.

[54] Gadamer H-G (2004). Truth and method.

[55] Statistics Norway. 11342: Area and population in municipalities, counties and the whole country (K) 2007 - 2020 2000 [Available from: https://www.ssb.no/statbank/table/11342. Accessed 1 Nov 2021.

[56] World Medical Association (2017). Ethical principles for medical research involving human subjects.

[57] Held V, Calhoun C (2004). Taking care: care as practice and value. Setting the moral compass: essays by women philosophers.

[58] Wulff J, Fänge AM, Lethin C, Chiatti C. Self-reported symptoms of depression and anxiety among informal caregivers of persons with dementia: a cross-sectional comparative study between Sweden and Italy. BMC Health Serv Res. 2020;20(1):1114. 10.1186/s12913-020-05964-2. 10.1186/s12913-020-05964-2 PMC7709414 33267856

[59] Liu S, Li C, Shi Z, Wang X, Zhou Y, Liu S, et al. Caregiver burden and prevalence of depression, anxiety and sleep disturbances in Alzheimer's disease caregivers in China. J Clin Nurs. 2017;26(9-10):1291–300. 10.1111/jocn.13601. 10.1111/jocn.13601 27681477

[60] Marasinghe MK. Assistive technologies in reducing caregiver burden among informal caregivers of older adults: a systematic review. Disabil Rehabil Assist Technol. 2016;11(5):353–60. 10.3109/17483107.2015.1087061. 10.3109/17483107.2015.1087061 26371519

[61] Plöthner M, Schmidt K, de Jong L, Zeidler J, Damm K. Needs and preferences of informal caregivers regarding outpatient care for the elderly: a systematic literature review. BMC Geriatr. 2019;19(1):82. 10.1186/s12877-019-1068-4 10.1186/s12877-019-1068-4 PMC6417014 30866827

[62] Karlsen C, Moe C, Haraldstad K, Thygesen E. Caring by telecare? A hermeneutic study of experiences among older adults and yheir family caregivers. J Clin Nurs. 2018;28(78):1300–13. 10.1111/jocn.14744. 10.1111/jocn.14744 30552788

[63] Malmgren Fänge A, Schmidt SM, Nilsson MH, Carlsson G, Liwander A, Dahlgren Bergström C, et al. The TECH@HOME study, a technological intervention to reduce caregiver burden for informal caregivers of people with dementia: study protocol for a randomized controlled trial. Trials. 2017;18(1):63. 10.1186/s13063-017-1796-8 10.1186/s13063-017-1796-8 PMC5301412 28183323

[64] Holthe T, Wulff-Jacobsen I. Matching user needs to technology in dementia care: experiences with the Alma supervisor educational program. Fam Med Prim Care Rev. 2016;18(4):492–6. 10.5114/fmpcr.2016.63710.

[65] Morley J, Floridi L. The limits of empowerment: how to reframe the role of mhealth tools in the healthcare ecosystem. Sci Eng Ethics. 2020;26(3):1159–83. 10.1007/s11948-019-00115-1 10.1007/s11948-019-00115-1 PMC7286867 31172424

[66] Stokke R. "maybe we should talk about it anyway": a qualitative study of understanding expectations and use of an established technology innovation in caring practices. BMC Health Serv Res. 2017;17. 10.1186/s12913-017-2587-3. 10.1186/s12913-017-2587-3 PMC5602870 28915809

[67] Nilsen E, Dugstad J, Eide H, Gullslett MK, Eide T. Exploring resistance to implementation of welfare technology in municipal healthcare services – a longitudinal case study. BMC Health Serv Res. 2016;16(1):1–14. 10.1186/s12913-016-1913-5. 10.1186/s12913-016-1913-5 PMC5111186 27846834

[68] Gheduzzi E, Masella C, Morelli N, Graffigna G. How to prevent and avoid barriers in co-production with family carers living in rural and remote area: an Italian case study. Res Involve Engage. 2021;7. 10.1186/s40900-021-00259-0. 10.1186/s40900-021-00259-0 PMC7968224 33731217

[69] Wolff JL, Freedman VA, Mulcahy JF, Kasper JD. Family caregivers’ experiences with health care workers in the care of older adults with activity limitations. JAMA Netw Open. 2020;3(1):e1919866. 10.1001/jamanetworkopen.2019.19866. 10.1001/jamanetworkopen.2019.19866 PMC6991279 31977063

[70] Heaton J, Day J, Britten N. Collaborative research and the co-production of knowledge for practice: an illustrative case study. Implement Sci. 2016;11(20):1–10. 10.1186/s13012-016-0383-9. 10.1186/s13012-016-0383-9 PMC4761144 26897169

[71] Glomsås HS, Knutsen IR, Fossum M, Halvorsen K. ‘They just came with the medication dispenser’- a qualitative study of elderly service users’ involvement and welfare technology in public home care services. BMC Health Serv Res. 2021;21:245.10.1186/s12913-021-06243-4. 10.1186/s12913-021-06243-4 PMC7977566 33740974

[72] United Nations. Universial declaration of human rights Paris1948. Available from: https://www.ohchr.org/EN/UDHR/Pages/Language.aspx?LangID=eng.

[73] Scott Kruse C, Karem P, Shifflett K, Vegi L, Ravi K, Brooks M. Evaluating barriers to adopting telemedicine worldwide: a systematic review. J Telemed Telecare. 2018;24(1):4–12. 10.1177/1357633X16674087. 10.1177/1357633X16674087 PMC5768250 29320966

